# Comprehension of acoustically degraded emotional prosody in Alzheimer’s disease and primary progressive aphasia

**DOI:** 10.1038/s41598-024-82694-z

**Published:** 2024-12-28

**Authors:** Jessica Jiang, Jeremy C. S. Johnson, Maï-Carmen Requena-Komuro, Elia Benhamou, Harri Sivasathiaseelan, Anthipa Chokesuwattanaskul, Annabel Nelson, Ross Nortley, Rimona S. Weil, Anna Volkmer, Charles R. Marshall, Doris-Eva Bamiou, Jason D. Warren, Chris J. D. Hardy

**Affiliations:** 1 Dementia Research Centre, Department of Neurodegenerative Disease, UCL Queen Square Institute of Neurology, University College London, 1st Floor, 8-11 Queen Square, London, WC1N 3AR UK; 2 Basic and Clinical Neuroscience, School of Neuroscience, King’s College London, London, UK; 3 Department of Psychology, Institute of Clinical Psychology and Psychotherapy Research, MSH Medical School Hamburg, Hamburg, Germany; 4 Division of Neurology, Department of Internal Medicine, King Chulalongkorn Memorial Hospital, Thai Red Cross Society, Bangkok, Thailand; 5 Cognitive Clinical and Computational Neuroscience Research Unit, Faculty of Medicine, Chulalongkorn University, Bangkok, Thailand; 6 Wexham Park Hospital, Frimley Health NHS Foundation Trust, Berkshire, UK; 7 Division of Psychology and Language Sciences, University College London, London, UK; 8 Centre for Preventive Neurology, Wolfson Institute of Population Health, Queen Mary University of London, London, UK; 9 UCL Ear Institute and UCL/UCLH Biomedical Research Centre, National Institute of Health Research, University College London, London, UK

**Keywords:** Degraded speech, Emotional prosody, Dementia, Primary progressive aphasia, Frontotemporal dementia, Alzheimer’s disease, Social cognition, Noise-vocoding, Auditory system, Cognitive neuroscience, Emotion

## Abstract

Previous research suggests that emotional prosody perception is impaired in neurodegenerative diseases like Alzheimer’s disease (AD) and primary progressive aphasia (PPA). However, no previous research has investigated emotional prosody perception in these diseases under non-ideal listening conditions. We recruited 18 patients with AD, and 31 with PPA (nine logopenic (lvPPA); 11 nonfluent/agrammatic (nfvPPA) and 11 semantic (svPPA)), together with 24 healthy age-matched individuals. Participants listened to speech stimuli conveying three emotions in clear and noise-vocoded forms and had to identify the emotion being conveyed. We then conducted correlation analyses between task performance and measures of socio-emotional functioning. All patient groups showed significant impairments in identifying clear emotional prosody compared to healthy individuals. These deficits were exacerbated under noise-vocoded conditions, with all patient groups performing significantly worse than healthy individuals and patients with lvPPA performing significantly worse than those with svPPA. Significant correlations with social cognition measures were observed more consistently for noise-vocoded than clear emotional prosody comprehension. These findings open a window on a dimension of real-world emotional communication that has often been overlooked in dementia, with particular relevance to social cognition, and begin to suggest a novel candidate paradigm for investigating and quantifying this systematically.

**Supplementary Information:**

The online version contains supplementary material available at 10.1038/s41598-024-82694-z.

## Introduction

Despite verbal content in speech being the main component of human communication for hearing individuals, there is much more to successful communication than just the verbal message. To understand the contextual form of the word and gain full insights into the speaker’s communicative intent, nonverbal auditory information also needs to be comprehended^1–3^. Prosody is a complex nonverbal speech attribute associated with acoustic properties of pitch, duration, intensity and their combination and modulation in intonation and stress patterns, over temporal scales ranging from individual phonemes to phrases. It carries multidimensional information and serves diverse functions, including disambiguating meaning and highlighting or emphasising particular elements in a spoken message, and signalling emotion^4^. Broadly, the functions of prosody can be considered linguistically (for example, to indicate whether a statement is declarative or interrogative), or affectively, to convey the speaker’s emotional state^5^. Modulations in vocal pitch (fundamental frequency (F0)), durational properties (such as syllable length), intensity, and voice quality perceived by listeners to convey emotional states are collectively known as ‘emotional prosody’^6,7^. Different emotions tend to create different prosody ‘profiles’ in speech. For instance, joy is typically characterised by a faster speech rate, higher intensity, and increases in F0 mean and variability, resulting in more melodic and energetic speech; while sadness is typically characterised by slower speech, at a lower intensity, with decreases in F0 mean and variability^8,9^. Impaired prosodic processing has been shown to have implications for social interactions and interpersonal relationships^6^.

Consideration of how processing of prosody is affected in neurodegenerative diseases is important, and may be particularly pertinent to language-led dementia (primary progressive aphasia, PPA) syndromes characterised by profound communication difficulties^10,11^. Syndromes across the Alzheimer’s disease (AD) and frontotemporal lobar degeneration spectrum are characterised by impairments in speech processing^12–16^ and/or social signal processing^17–21^. Previous research has identified impairments in nonverbal auditory perception in typical AD and most notably, in non-fluent/agrammatic variant PPA (nfvPPA) and semantic variant PPA (svPPA)^22–27^. Impaired perception of emotional prosody has been documented in typical AD^7,28–31^, svPPA, nfvPPA and the language variant of AD, logopenic variant PPA (lvPPA)^5,32–34^. Patients with lvPPA have impaired tracking of an acoustic parameter (speech envelope) relevant to prosody perception^35^.

However, all these studies have considered emotional prosodic comprehension using ‘clear’ speech stimuli under ideal, laboratory settings, which are unlikely to reflect the reality of communication in daily life, where speech is often degraded in quality or masked by noise and other competing signals. The comprehension of ‘degraded’ emotional prosody comprehension has been largely unexplored. One widely used technique for altering speech signals experimentally is noise-vocoding, whereby the speech signal is divided digitally into discrete frequency bands (‘channels’), each filled with white noise and modulated by the amplitude envelope of the original signal^36^. Noise-vocoding approximately simulates the common everyday experience of interpreting vocal signals with reduced acoustic quality (such as over a low-quality telephone or video connection or when whispered)^37^. Among various alternative methods^37,38^, noise-vocoding is an attractive paradigm to study the effects of neurodegenerative diseases on the comprehension of degraded speech and in particular vocal emotional signals, for three main reasons. Firstly, it allows for parameterisation of the amount of acoustic degradation of a speech signal. Secondly, spectral detail is important to many emotional prosodic cues; such cues are therefore likely to be sensitive to noise-vocoding, as a technique that targets spectral detail in speech signals. Thirdly, noise-vocoding has previously been used successfully to extract thresholds for comprehension of degraded verbal messages in the diagnostic groups targeted in the present study^39^, suggesting that this paradigm might be well adapted for deriving analogous measures for degraded prosodic comprehension in the same clinical populations.

Here, we explored perception of emotional prosody in major PPA syndromes and AD, both in ‘clear’ and ‘degraded’ speech forms. In line with previous research^5,7,28–35,40^ and our own cumulative clinical experience, we hypothesised that people with AD and PPA would perform worse than healthy age-matched individuals at identifying ‘clear’ emotional prosody, with an additional performance cost from degrading emotional prosodic cues; and that these deficits would be most marked in nfvPPA and lvPPA^39^. We further predicted that emotional prosody comprehension performance would correlate with measures of daily life socio-emotional functioning.

## Materials and methods

### Participants

Eighteen patients with typical amnestic AD, nine patients with lvPPA, 11 patients with nfvPPA, and 11 patients with svPPA were recruited via a specialist cognitive clinic. All patients fulfilled consensus clinical diagnostic criteria^14,41^. Twenty-four healthy age-matched individuals with no history of neurological or psychiatric disorders were recruited from the Dementia Research Centre volunteer database.

All participants had a comprehensive general neuropsychological assessment (Table 1). None had a history of otological disease, other than presbycusis; participants assessed in person at the research centre had their peripheral hearing assessed using pure-tone audiometry, following a previously described procedure (details in Supplementary Material is available online). No participants were excluded based on their general neuropsychological testing performance.

**Table 1:** General demographic, clinical and neuropsychological characteristics of all participant groups.

Characteristic	Healthy Individuals	AD	lvPPA	nfvPPA	svPPA
Demographic and clinical					
No. M: F	13:11	13:5	7:2	8:3	7:4
Age, years	68.7 (6.40)	70 (8.86)	70.4 (6.46)	68.7 (6.05)	62.5 (8.57)
Handedness (R: L:A)	21:1:1^a^	17:1:0	9:0:0	11:0:0	10:1:0
Education (y)	16.1 (2.72)^a^	15.4 (3.89)^a^	14.6 (2.72)^a^	14.6 (2.72)^a^	15.7 (2.11)^a^
Symptom duration (y)	NA	6 (3.10)^a^	5.75 (4.53)^a^	3.36 (1.57)	5.45 (2.62)
Best ear average*	17.9 (10.3)^b^	28.1 (12.6)^h^	17.5 (12.9)^j^	30 (4.18)^k^	22.7 (8.64)^l^
Tested in-person/remote	20:4	9:9	5:9	6:5	4:7
cMMSE (/30)	29.48 (1.25)^c^	**21 (5.66)** ^c^	**22.25 (6.54)** ^a^	**25.55 (3.27)** ^1^	**24.82 (4.40)** ^1^
General neuropsychology					
Executive function					
WASI Matrices (/32)	26.8 (2.74)^f^	**12.4 (8.58)** ^**i**^	**21.9 (5.18)**	**22.1 (6.89)** ^**1**^	25.5 (5.01)^1^
Letter fluency (total)	15.9 (5.35)^f^	10.9 (5.86)^a^	**8.22 (4.21)**	9.11 (8.43)^i^	**7.73 (6.62)**
Category fluency (total)	24.1 (6.30)^f^	**11.4 (6.74)** ^**a**^	**10.6 (6.19)**	16.8 (8.97)^2,i^	**7.27 (5.57)**
Working memory					
Digit span forward (max)	6.56 (1.03)^f^	5.78 (1.44)	**4.33 (1.32)** ^**2**^	**4.91 (1.76)** ^**2**^	6.64 (1.03)
Digit span reverse (max)	5.19 (1.17)^f^	**3.28 (1.41)**	**3.22 (1.72)** ^**2**^	**3.55 (1.69)** ^**1,2**^	5.00 (1.61)^1^
Auditory input processing					
PALPA-3 (/36)	34.6 (1.66)^g^	NA	33.5 (2.39)^a^	32.9 (2.63)	33.6 (2.38)
Language skills					
GNT (/30)	25.8 (2.46)^f^	**13.2 (7.39)**	**9.67 (7.43)**	**17.7 (10.8)** ^**1**^	**1.55 (4.50)** ^**1**^
BPVS (/150)	148 (2.09)^f^	**139 (15.6)**	141 (13.2)^2^	**135 (19.1)** ^**2**^	**79.8 (48.1)** ^**1**^
PALPA-55 (/24)	23.5 (1.20)^g^	NA	**17.00 (7.01)** ^**a**^	**19.3 (3.58)**	**19.9 (5.05)**
Modified Camel and Cactus (/32)	30.6 (1.09)^f^	NA	**26.4 (7.60)** ^**a**^	28.5 (3.36)^2^	**21.5 (7.92)** ^**a**^
Episodic memory					
RMT Faces (Short) (/25)	23.8 (2.5)^c^	**16.1 (3.26)** ^**h**^	21.4 (3.65)^l^	22.8 (3.49)^1,j^	19.2 (3.69)^m^
RMT Faces (Long) (/50)	41.7 3.70)^e^	**29.6 (5.57)** ^**h**^	**28.5 (5.74)** ^**i**^	36.8 (7.27)^m^	**31.1 (3.53)** ^**l**^
Other skills					
GDA calculation (/24)	14.8 (5.18)^f^	**5.06 (4.81)** ^**i**^	**5.38 (4.63)** ^**2,a**^	**6.09 (5.41)**	11.2 (5.67)^1,a^
VOSP Object Decision(/20)	18.9 (1.48)^f^	**14.6 (2.94)**	**16.2 (2.49)** ^**a**^	17.9 (1.85)^1,a^	**15.7 (3.95)** ^**a**^
Social Cognition Questionnaire Measures					
mIRI: total	NA	40.9 (12.2)	40.2 (7.27)	42.9 (7.93)	38.3 (13.9)
Cognitive Empathy	NA	18.3 (6.63)	18.4 (3.46)	19 (3.37)	16.5 (6.47)
Emotional Empathy	NA	22.6 (6.80)	21.9 (4.39)	23.9 (5.43)	21.8 (8.08)
RSMS: total	NA	32.9 (7.03)	32.8 (9.21)	37.2 (13.0)	29.5 (13.8)
Sensitivity to Expressive Behaviour	NA	15.3 (4.64)	14.2 (5.55)	16.8 (8.89)	12.3 (8.33)
Monitor Self-Presentation	NA	17.6 (4.39)	18.5 (4.04)	22 (5.83)	17.2 (6.08)

Mean (standard deviation) values and raw scores are presented (maximum value possible in parentheses), unless otherwise indicated; full details of the remote neuropsychological test battery are given in^42^. Significant differences from healthy individuals (*p* < 0.05) are in bold;

A, ambidextrous; AD, patient group with typical Alzheimer’s disease; BNT, Boston Naming Test; BPVS, British Picture Vocabulary Scale; cMMSE, combined Mini-Mental State Examination score (derived for participants completing either in-person MMSE or tele-MMSE – see text); Digit span forward/reverse, maximum digit span recorded; F, female; GDA, Graded Difficulty Arithmetic; GNT, Graded Naming Test; L, left; lvPPA, patient group with logopenic variant primary progressive aphasia; M, male; mIRI, modified Interpersonal Reactivity Index; NA, not available/applicable; nfvPPA, patient group with nonfluent/agrammatic variant primary progressive aphasia; PALPA, Psycholinguistic Assessments of Language Processing in Aphasia; R, right; RMT, Recognition Memory Test (the RMT Faces (Long) was administered to participants in-person, while the RMT Faces (Short) was administered to participants remotely); RSMS, Revised Self-Monitoring Scale; svPPA, patient group with semantic variant primary progressive aphasia; Synonyms concrete/abstract, single-word comprehension of single words; T-MMSE, tele-MMSE; VOSP, Visual Object and Space Perception battery; WASI, Wechsler Abbreviated Scale of Intelligence.

^1^significantly different to AD (*p* < 0.05);

^2^significantly different to svPPA (*p* < 0.05). *See Supplementary Material for details concerning the ‘best ear average’ measure. Numbers of participants for which data are missing for particular tests are coded as follows: ^a^one participant; ^b^14 participants; ^c^3 participants; ^d^15 participants; ^e^20 participants; ^f^8 participants; ^g^11 participants; ^h^9 participants; ^i^2 participants; ^j^5 participants; ^k^6 participants; ^l^4 participants; ^m^7 participants.

Due to the COVID-19 pandemic, some data for this study were collected remotely (see Supplementary Materials). We have described the design and implementation of our remote neuropsychological assessment protocol elsewhere^42^. Participants assed remotely completed the T-MMSE^43^ while those assessed in-person completed the standard MMSE; for all participants, we applied a scalar conversion to generate a combined MMSE score for incorporation into analyses^43^ (Table 1). Performance profiles of seven healthy control participants who performed the experiment both in person and subsequently remotely were very similar, justifying combining participants tested in person and remotely in the main analyses (Fig. S3)^39^.

All participants gave written informed consent to take part in the study. Ethical approval was granted by the UCL-NHNN Joint Research Ethics Committees, in accordance with Declaration of Helsinki guidelines.

### Creation of experimental stimuli

Forty-five three-digit numbers (of the form: ‘three-hundred-and-seventy-three’), spoken by two adult male and two adult female speakers (all with Standard Southern British English accents), were taken from a previously normed set of vocal emotional stimuli^44^. The full stimuli set comprises six emotions (namely anger, surprise, sadness, fear, disgust and happiness). Here, to reduce response demands for patients, we selected numbers portraying three emotions: anger, surprise and sadness, chosen because previous work has shown that healthy older individuals are consistently able to identify these^5^.

Speech recordings were noise-vocoded using Praat (https://www.fon.hum.uva.nl/praat/) to generate acoustically altered stimuli at either six, 12 or 18 channels (see Supplementary Fig. 1 for spectrograms). Details concerning the synthesis of noise-vocoded stimuli are provided in Supplementary Material online. Three levels of noise-vocoding were used due to considerations of the length of the task and concerns over participant fatigue, in comparison to a previous thresholding procedure which included 24 vocoding channels^39^. The three channels chosen were designated to signify hard (six channels), medium (12 channels) and easy (18 channels) comprehension. This was informed through previous work showing that vocoded speech at 10 channels is readily intelligible by healthy listeners, whereas four channels only becomes intelligible after hours of training^45^. At each noise-vocoding level, 15 three-digit number stimuli were presented, with five numbers in each of the three emotions. Twenty-one three-digit number stimuli were kept in ‘clear’ form: six (two for each emotion) were used as practice items for familiarising the participant with the stimuli before the experimental test, and 15 (five per emotion) were used as a clear speech control condition. Thus, a total of 60 stimuli (20 for each emotion) were presented during the experimental test session, which typically lasted around 20 min.

### Experimental procedure

The stimuli were administered either in-person in a quiet room via Audio-Technica ATH-M50x headphones at a comfortable fixed listening level (at least 70 dB), or remotely via Labvanced and shared through a video link (see Supplementary materials online).

To be familiarised with the experimental procedure, participants first heard the six practice clear three-digit number stimuli, and asked to identify which emotion each number was spoken with, using a cue card as a guide and response aid if necessary (Fig. S2). Feedback was given for the practice trials and participants were told if they had answered correctly on each of the practice trials.

Participants then continued to the experimental task proper, where first, the 45 noise-vocoded speech trials were presented in randomised order to each participant, followed by the 15 clear speech trials, again in randomised order. On each trial, participants were tasked to indicate, either verbally or by pointing at the cue card (Fig. S2), which emotion was portrayed (e.g., was it sadness, anger or surprise? ). Responses were noted by the examiner for offline analysis, no feedback about the performance was given, and no time limits were imposed.

### Assessment of social cognition

To assess patients’ social cognition, the modified Interpersonal Reactivity Index (mIRI) and the Revised Self-Monitoring Scale (RSMS) questionnaires were completed by the primary caregiver or another close informant on each patient’s behalf.

The mIRI, frequently used with people with dementia^46,47^, is based on the Interpersonal Reactivity Index^48^. It includes two seven-item subscales: the first subscale measures cognitive empathy in the form of perspective taking, and the second subscale assesses emotional empathy in the form of empathic concern. The questions are formatted in a series of statements and responders are asked how well each statement describes the participant on a Likert response scale.

The RSMS (Lennox & Wolfe, 1984), also frequently used with people with dementia^49,50^, is a 13-item questionnaire based on the Self-Monitoring Scale^51^. It is made up of two subscales: the first subscale measures participants’ sensitivity to expressive behaviour, and the second subscale measures the tendency to monitor self-presentation. The questions are formatted in a series of statements and responders are asked how well each statement describes the participant on a Likert response scale.

### Data analysis

Data were analysed in R^®^ (v4). For continuous demographic and background neuropsychological data, participant groups were compared using ANOVA or Kruskal Wallis tests, depending on whether assumptions of the general linear model were met. Group categorical data were compared using Fisher’s exact test.

For the main experimental and control (i.e. emotional prosody) tests, a binary score was given for each trial: one if the emotion was correctly identified, zero if incorrect. To reduce bias in subsequent analyses, participants who scored at or below chance on the clear emotion recognition control task (i.e., a score that could have been achieved by random guessing) were excluded. A threshold for chance performance was calculated using the cumulative probability function: 15 trials with probability 0.33’ suggested that a hit rate (k) of nine (out of 15) or above was unlikely to be achieved by chance (*p* = 0.029 [k = 8, *p* = 0.084])^52^.

For the control task (clear emotion comprehension), due to ceiling effects, data were analysed using the Kruskal Wallis test. For the experimental task, data were analysed using a mixed ANCOVA, with vocoding channels as the repeated measure (three levels: six [hard], 12 [medium], 18 [easy]) and diagnosis as the between-subjects factor, adjusting for performance on the control task (performance on clear emotions), and Wechsler Abbreviated Scale of Intelligence (WASI) Matrix Reasoning (as a proxy for disease severity). Additionally, the model included an interaction term between diagnosis and vocoding channel number. Where the omnibus test was significant, post hoc analyses were conducted (pairwise t-tests for ANCOVA; Dunn test for Kruskal Wallis). To facilitate comparison with the control task performance, we also ran unadjusted Kruskal Wallis tests on vocoded task performance at each channel level separately.

Spearman’s rank correlation was used to assess the relationship between accurate comprehension of degraded emotional prosody and disease severity (WASI matrix reasoning), auditory perception (Psycholinguistic Assessment of Language Processing in Aphasia (PALPA)-3, pure-tone audiometry), working memory (digit span), and measures of socio-emotional awareness (mIRI and RSMS). To assess whether any significant correlations between noise-vocoded emotional prosody comprehension and socio-emotional awareness could be explained by generic noise-vocoded speech perception ability rather than specific degraded emotional prosodic comprehension skills, we additionally conducted correlation analyses with performance on a neutral prosody noise-vocoded number repetition task (from^39^) as the majority of participants had also completed this (excluding three patients with nfvPPA)).

Considering the small cohort sizes and exploratory nature of the study, no corrections for multiple comparisons were conducted, to avoid inflating type II error. Effect sizes (epsilon squared for Kruskal Wallis models (ε^2^); partial eta squared for ANCOVA models (η^2^_p_); Spearman’s rank correlation coefficient (rs) were generated in addition to p-values, and an alpha of 0.05 was adopted as the threshold for statistical significance on all tests.

## Results

General neuropsychology profiles were in keeping with the syndromic diagnosis for each patient group (Table 1).

Participants who scored at or below chance on the clear emotion comprehension control task were excluded from subsequent analyses (two patients with AD, one with nfvPPA, and one with svPPA). An additional AD patient was excluded as they were unable to correctly identify any of the ‘angry’ stimuli in the clear speech condition. Sixty-eight participants were included in the final analyses.

### General participant group characteristics

Participant groups did not differ significantly in age, sex, handedness, years of formal education or pure-tone audiometry (all *p* > 0.05). Patient groups did not differ in mean symptom duration (*p* = 0.136) or combined MMSE score (*p* = 0.069). Basic speech discrimination (assessed using the PALPA-3^53^) performance also did not differ significantly across participant groups (*p* = 0.366).

### Experimental behavioural data

**Fig. 1:**
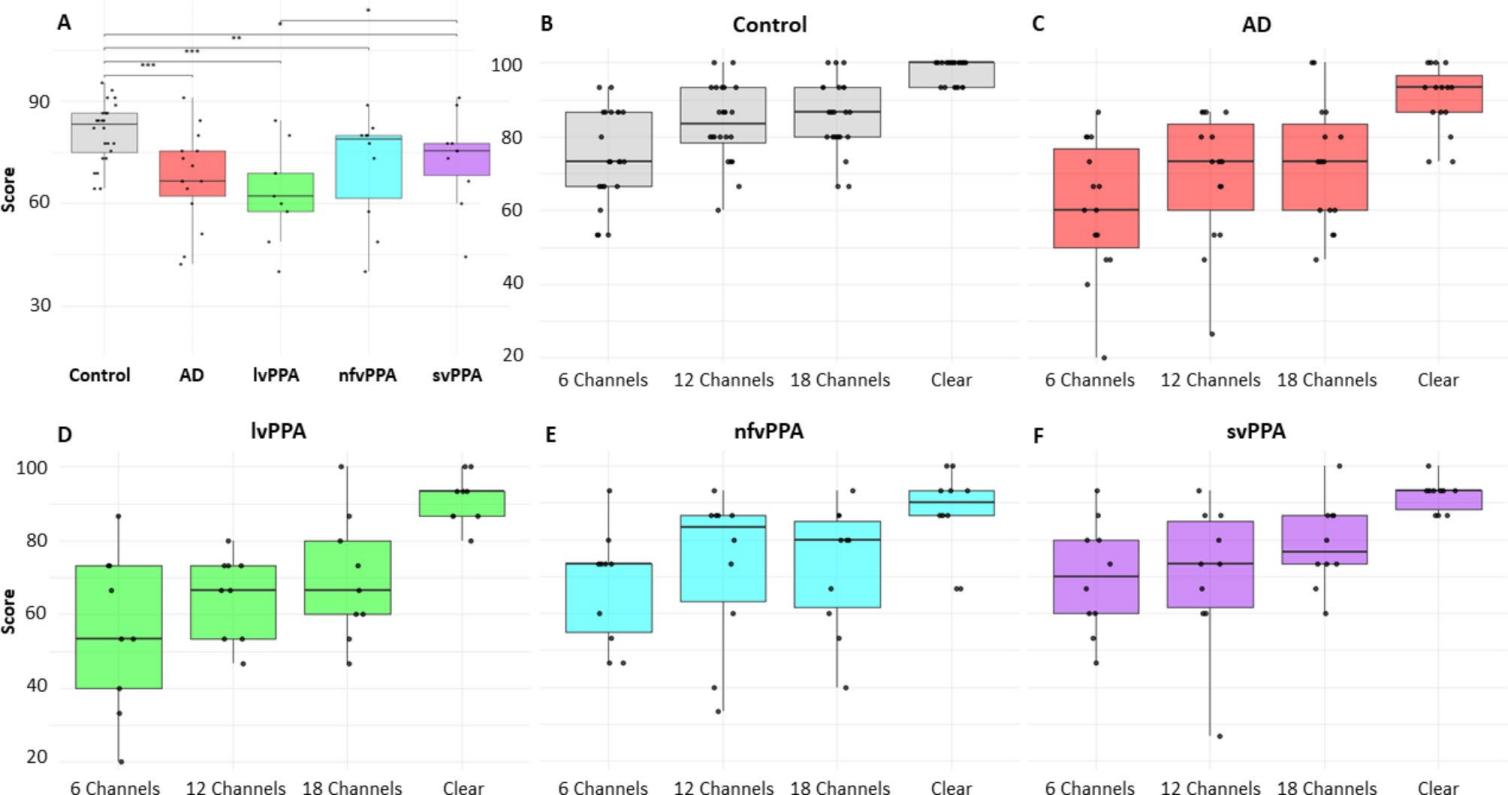
Boxplots of noise-vocoded emotional comprehension performance for each diagnostic group. Panel A shows data across participant groups; here, score refers to the percentage correct of the combined noise-vocoded prosody comprehension score, across all three vocoding channel levels. Significant between-group differences after adjusting for performance on identifying clear emotions and WASI matrix reasoning (as an index of disease severity) are coded with *** as *p* < 0.001, ** as *p* < 0.01 and * as *p* < 0.05. Panels B - F show performance profiles across vocoding channels (and in clear speech) for each participant group separately; score refers to the percentage correct of the prosody comprehension performance at each vocoding level. In all panels, the horizontal line within each box indicates the median score, with the boxes indicating the interquartile range; individual participant data points are superimposed. AD, patient group with typical Alzheimer’s disease; Control, healthy age-matched individuals; lvPPA, patient group with logopenic variant primary progressive aphasia; nfvPPA, patient group with nonfluent/agrammatic variant primary progressive aphasia; svPPA, patient group with semantic variant primary progressive aphasia.

Data for comprehension of clear and vocoded emotional prosody for all participant groups are summarised in Table 2; Fig. 1.

**Table 2:** Mean correct raw scores for comprehension of emotional prosody in clear and noise-vocoded speech, in each participant group.

		Healthy individuals	AD	lvPPA	nfvPPA	svPPA
Correct Raw Responses						
Clear (/15)		14.71 (0.46)	**13.53 (1.36)**	**13.67 (1.36)**	**13.10 (1)**	**13.80 (0.63)**
Vocoded	**18 Channels (/15)**	12.83 (1.40)	**10.87 (2.50)**	**10.44 (2.56)**	**10.90 (2.56)**	11.80 (1.75)
	**12 Channels (/15)**	12.50 (1.53)	**10.40 (2.64)**	**9.78 (1.72)**	10.90 (3.18)	**10.60 (2.88)**
	**6 Channels (/15)**	11.13 (1.85)	**9.13 (2.72)**	**8.33 (3.24)**	10.10 (2.28)	10.50 (2.27)
	**Combined (/45)**	36.46 (3.98)	**30.40 (6.23)**	28.56 (6.33)*	**31.90 (7.29)**	**32.90 (6.12)**

Mean (standard deviation) values are shown. Raw scores are presented (maximum value possible in parentheses). Values significantly different from healthy age-matched individuals (*p* < 0.05) are in **bold** and from svPPA noted with *. For clear and vocoded performance at separate channel levels, significant differences were generated using unadjusted Kruskal Wallis tests. For the combined vocoded performance across all channel levels, the model used was a mixed ANCOVA, with vocoding channels as the repeated measure (three levels: six [hard], 12 [medium], 18 [easy]) and diagnosis as the between-subjects factor, adjusting for performance on the control task (performance on clear emotions), and Wechsler Abbreviated Scale of Intelligence (WASI) Matrix Reasoning (as a proxy for disease severity).

AD, patient group with typical Alzheimer’s disease; lvPPA, patient group with logopenic variant primary progressive aphasia; nfvPPA, patient group with nonfluent variant primary progressive aphasia; svPPA, patient group with semantic variant primary progressive aphasia.

There was a significant difference between diagnostic groups on comprehension of the clear emotional stimuli (Table 2) (χ2(4) = 20.22, *p* = 0.001, ε^2^ = 0.24, 95% CI [0.13, 0.46]), with all patient groups performing worse than healthy individuals (AD: t=-3.23, *p* = 0.001; lvPPA: t=-2.86, *p* = 0.004; nfvPPA: t=-3.40, *p* < 0.001; svPPA t=-2.93, *p* = 0.003) (Fig. S4). There were no differences between patient groups (*p* > 0.05).

After adjusting for performance on identifying clear emotions and WASI matrix reasoning (as an index of disease severity), there was a significant main effect of diagnosis on vocoded emotional prosody comprehension (F(4,58) = 5.47, *p* < 0.001, η^2^_p_ = 0.27, 95% CI [0.09, 1.00]). There were also significant effects of clear emotion comprehension performance (F(1,58) = 8.45, *p* = 0.005, η^2^_p_ = 0.13, 95% CI [0.02, 1.00]), vocoding channels (F(2,120) = 18.03, *p* < 0.001, η^2^_p_ = 0.23, 95% CI [0.12, 1.00]), and WASI matrix reasoning (F(1,58) = 4.46, *p* = 0.039, η^2^_p_ = 0.07, 95% CI [0.00, 1.00]). Post-hoc analyses showed that all patient groups performed worse than the healthy individuals (all *p* < 0.03), and the lvPPA patient group performed significantly worse than the svPPA group (t=-2.35, *p* = 0.020); but no other between-group comparisons were significant (all *p* > 0.05) (Table 2; Fig. 1).

Across groups, participants performed significantly worse at six channels compared with 12 (t = 2.63, *p* = 0.009) and 18 (t = 3.72, *p* < 0.001) channels (Fig. 1). There was no significant difference between performance at 12 and 18 channels (t = 1.08, *p* = 0.280). The interaction between diagnosis and vocoding channel number was also not significant (F(8,120) = 0.60, *p* = 0.774).

### Correlational analyses

In the combined patient cohort, performance on noise-vocoded emotional prosody comprehension was not significantly correlated with peripheral hearing (as measured with pure-tone audiometry; rs(21)=-0.09, *p* = 0.669) or speech discrimination (as measured with the PALPA-3; rs(26) = 0.23, *p* = 0.240)). Noise-vocoded emotional prosody comprehension was significantly correlated with WASI matrix reasoning score (rs(40) = 0.34, *p* = 0.029), forward digit span (rs(42) = 0.44, *p* = 0.003) and reverse digit span (rs(42) = 0.36, *p* = 0.018).

**Fig. 2:**
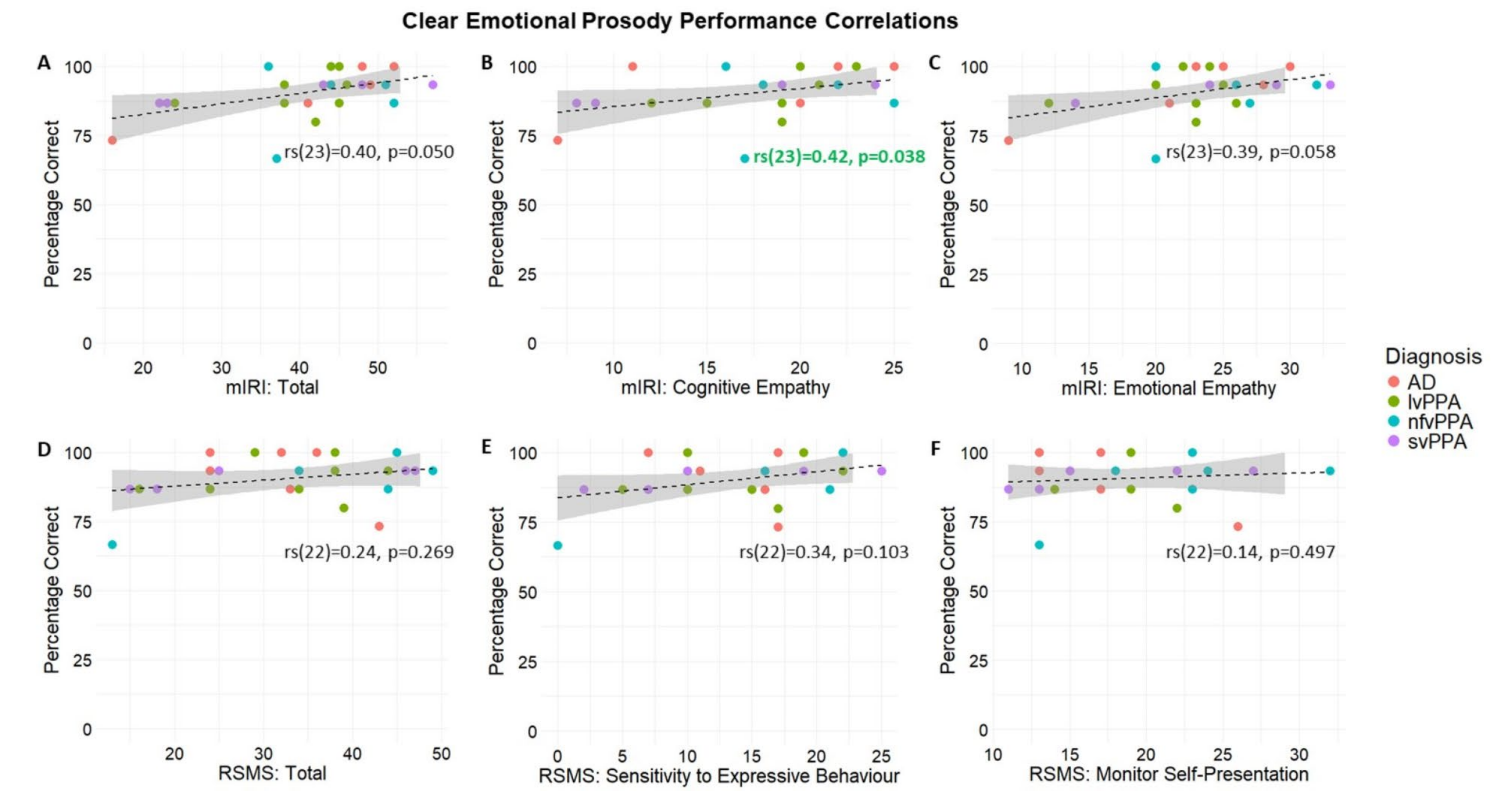
Correlation plots of clear emotional prosody comprehension with measures of social cognition across the patient cohort. This Figure shows how different standard measures of social cognition were correlated with total score on recognition of clear (natural) emotional prosody (see text) across syndromic groups, as follows: (**A**) correlation with the full modified Interpersonal Reactivity Index (mIRI); (**B**) correlation with the cognitive empathy subscale in mIRI; (**C**) correlation with the cognitive empathy (perspective taking) subscale in mIRI; (**D**) correlation with the full revised self-monitoring scale (RSMS); (**E**) correlation with the sensitivity to socio-emotional expressiveness RSMS subscale; (**F**) correlation with the monitoring self-presentation RSMS subscale. Spearman’s rank and p-value shown alongside each correlation line, **bold** green font indicates a significant correlation (*p* < 0.05). The percentage correct here is the percentage correct for clear emotional prosody (control task). Dots represent individual participants’ performance, with colours representing each syndromic diagnosis, as coded in the key (right); shading represents 95% confidence intervals. AD, Alzheimer’s disease; lvPPA, logopenic variant primary progressive aphasia; mIRI, modified Interpersonal Reactivity Index; nfvPPA, nonfluent variant primary progressive aphasia; RSMS, revised self-monitoring scale; svPPA, semantic variant primary progressive aphasia.

**Fig. 3:**
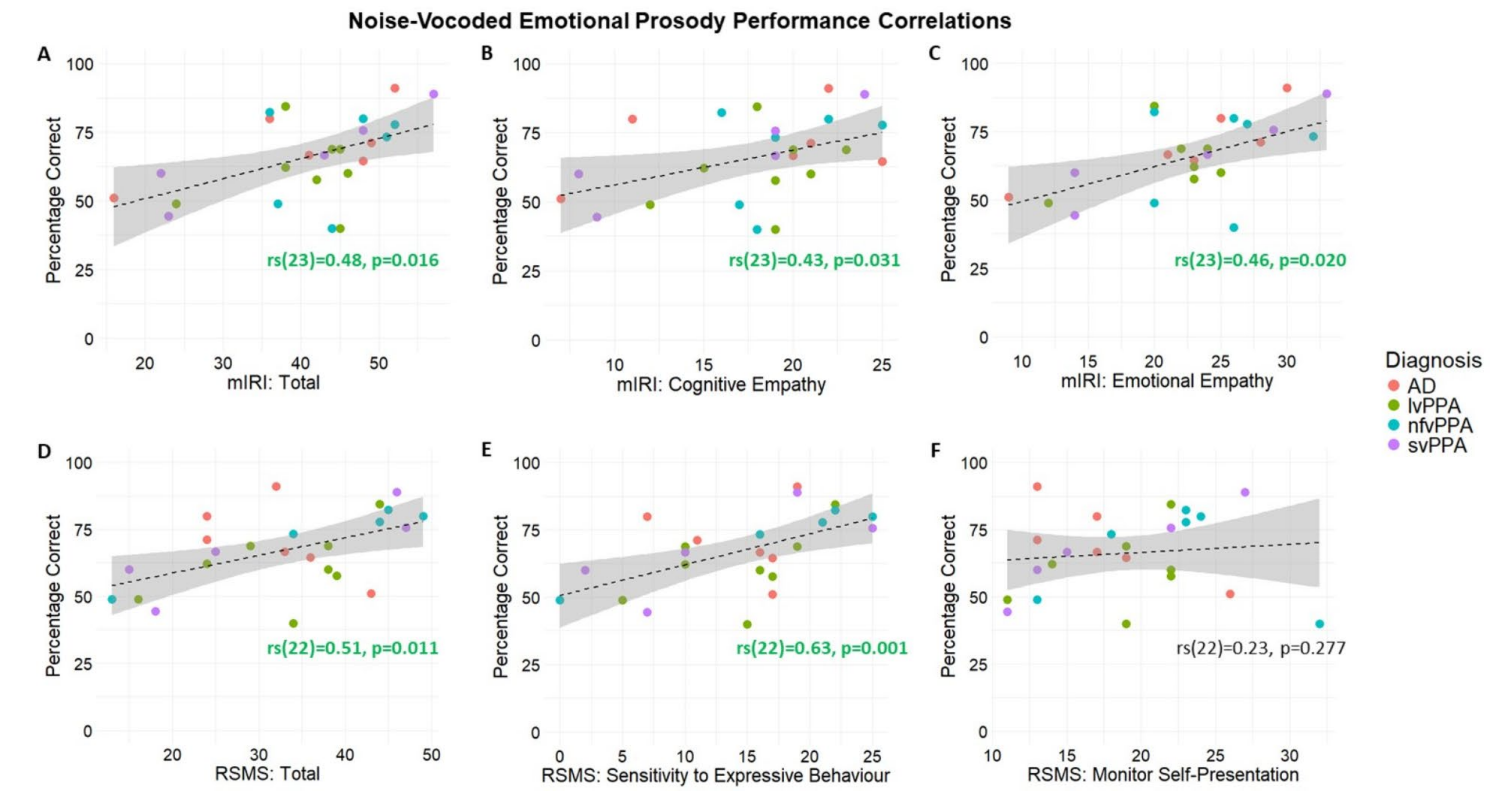
Correlation plots of noise-vocoded emotional prosody comprehension with measures of social cognition across the patient cohort. This Figure shows how different standard measures of social cognition were correlated with total score on recognition of noise-vocoded emotional prosody (see text) across syndromic groups, as follows: (**A**) correlation with the full modified Interpersonal Reactivity Index (mIRI); (**B**) correlation with the cognitive empathy subscale in mIRI; (**C**) correlation with the cognitive empathy (perspective taking) subscale in mIRI; (**D**) correlation with the full revised self-monitoring scale (RSMS); (**E**) correlation with the sensitivity to socio-emotional expressiveness RSMS subscale; (**F**) correlation with the monitoring self-presentation RSMS subscale. Spearman’s rank and p-value shown alongside each correlation line, **bold** green font indicates a significant correlation (*p* < 0.05). The percentage correct here is the combined noise-vocoded score (combined across all three levels of vocoding channels: see text). Dots represent indvidual participants’ performance, with different colours representing each syndromic diagnosis, as coded in the key (right); shading represents 95% confidence intervals. AD, Alzheimer’s disease; lvPPA, logopenic variant primary progressive aphasia; mIRI, modified Interpersonal Reactivity Index; nfvPPA, nonfluent variant primary progressive aphasia; RSMS, revised self-monitoring scale; svPPA, semantic variant primary progressive aphasia.

Total scores on the clear and noise-vocoded emotional prosody tasks were also significantly correlated with relevant scores on the mIRI and RSMS across the combined patient cohort (Figs. 2 and 3, respectively). Accurate comprehension of clear emotional prosody was significantly correlated with the mIRI subscale: cognitive empathy (perspective taking) (*p* = 0.038) and at threshold for significance with total mIRI score (*p* = 0.050) (Fig. 2). Accurate comprehension of noise-vocoded emotional prosody was significantly correlated with the full mIRI (*p* = 0.016), mIRI subscale: emotional empathy (*p* = 0.020), mIRI subscale: cognitive empathy (*p* = 0.031), the full RSMS (*p* = 0.011), and the RSMS subscale: sensitivity to expressive behaviour (*p* = 0.001; Fig. 3).

No significant correlations were observed between noise-vocoded number repetition (i.e. a non-prosodic degraded speech control) and any of the social cognition (mIRI or RSMS) measures (all *p* > 0.05) (Fig. S5).

## Discussion

Here, we have shown that comprehension of acoustically degraded emotional prosody is impaired in patients with AD and PPA syndromes relative to healthy age-matched individuals. In line with previous research and our hypotheses, we showed consistent deficits in clear prosody perception^5,28,34^. Furthermore, the deficit for emotional prosody comprehension in noise-vocoded speech remained after adjusting for performance on the ‘clear’ (i.e. natural, undistorted) speech task in the present study, suggesting that deficits in emotional prosody comprehension are exacerbated in non-ideal listening conditions in patients with PPA and AD. Based on previous research suggesting that patients with lvPPA and nfvPPA may be particularly susceptible to the effects of noise vocoding^39^, we had hypothesised that this additional ‘cost’ to noise-vocoding would be more apparent in these groups. However, all of the patient groups had significantly greater costs to noise-vocoding compared with the healthy controls, and the only between-patient group significant contrast was between lvPPA and svPPA. These findings could potentially reflect separate mechanisms, including previously identified problems with perceiving noise-vocoded auditory stimuli^39^, a core deficit in AD and lvPPA for apperceptive processing (e.g., the representation and decoding of auditory objects), and impaired disambiguation of degraded speech signals in svPPA^54^. There are likely to be stored neural ‘templates’ corresponding to the perceptual characteristics of the prosodic signatures of particular emotions, and under acoustic degradation, the neural template matching is stressed, analogous with previous work in AD and lvPPA suggesting impaired ‘template activation’ for phonemes^55–57^.

We also identified significant associations between emotional prosody comprehension performance and scores on two social cognition questionnaires (mIRI and RSMS) and their subscales, more consistently for vocoded than clear stimuli. In clear emotional prosody, there was a significant correlation with the mIRI cognitive empathy subscale and a borderline significant correlation with the full mIRI (Fig. 2). For noise-vocoded emotional prosody, we identified the same significant correlations as with clear emotional prosody comprehension performance and found additional significant correlations with the mIRI emotional empathy subscale, the full RSMS and the RSMS sensitivity to expressive behaviour subscale (Fig. 3). Patient groups did not differ significantly in mean scores on any of the social cognition measures assessed here, although patients with svPPA did tend to have lower scores than those with other diagnoses, consistent with previous research suggesting that social cognition is impaired in this population^10,58^. There was also considerable individual heterogeneity within groups on the mIRI and RSMS (and subscales) (see Table 1), which should be taken into consideration when interpreting the results of the correlation analyses; previous studies incorporating these measures have recorded similar degrees of variability^47,50^. Importantly, no significant correlations were identified between social cognition questionnaire scores and a noise-vocoded number repetition control task (Fig. S5), nor between noise-vocoded emotional prosody comprehension and pure-tone audiometry or speech discrimination task performance, implying that dissociable, central auditory mechanisms process verbal and nonverbal dimensions of speech signals. We did observe significant correlations between noise-vocoded nonverbal emotional prosody comprehension and measures of executive functioning and working memory (i.e. WASI Matrix Reasoning and digit span tests), consistent with previous research implicating the involvement of a fronto-parietal brain network^59–61^ in these processes.

We chose to use the paradigm of noise-vocoding in the current study as a model to simulate challenging listening conditions relevant to those encountered in daily life (such as a poor quality telephone or internet connection). Our findings have potential clinical significance: noise-vocoding may represent a ‘stress test’ of vocal emotion comprehension by patients with dementia in suboptimal everyday listening environments. Further, as comprehension of noise-vocoded vocal emotions correlates with measures of social cognition, this paradigm might be developed to generate clinical markers of social cognitive impairment. The present results corroborate previous evidence that noise-vocoding is a clinically relevant procedure for assessing the impact of acoustic degradation on the extraction of different kinds of information from verbal messages by listeners with dementia^39^. One possible explanation is that patients with neurodegenerative diseases targeting core auditory processing networks experience difficulties comprehending emotional cues within our daily hearing environments (i.e. an intrinsic auditory deficit), meaning that they are therefore less likely to engage with emotional cues being spoken and utilised towards them, manifesting as perceived socio-emotional difficulties which are actually secondary to the more basic auditory deficit^39,54,62^. An alternative explanation is that patients with these conditions have a ‘double-hit’ of a top-down impairment in social cognition^58,63,64^ coupled with the bottom-up auditory processing deficit. Either way, the significant correlation here implies that our noise-vocoded emotional prosody comprehension task could hold promise as a tool for tracking impairment.

There are several limitations in this experiment and more work needs to be conducted to not only refine the paradigm used here but to also understand the underlying mechanisms, as well as how they are impacted in different dementias and implications for daily life function in patients. Firstly, across groups, participants in the present study performed significantly worse at six than 12 channels, and at six than 18 channels, but there were no significant differences between 12 and 18 channels. This ‘flattening’ between 12 and 18 channels is perhaps unsurprising as it is consistent with the nonlinear noise-vocoding scale (e.g., the perceptual difference between 10 and 11 channels is negligible, whereas the difference between one and two channels is drastic), but future work should aim to refine channel selection. Secondly, the group sizes reported here were relatively small. Considering the rarity of PPA, the collection of substantially larger datasets would require multi-centre collaboration. Thirdly, while we deliberately selected spoken numbers as the prosodic ‘carrier’ in order to reduce potential top-down effects from semantic content (a strategy previously used successfully in studies of both cognitively impaired and normal listeners:^5,44^), in daily life it is unusual for numbers to be spoken with the emotions studied here, which may affect the generalizability of our findings. Fourthly, and relatedly, noise-vocoding was employed as this allowed us to tightly control the degree of degradedness of the stimuli; while there are qualitative similarities between noise-vocoding and certain daily-life communication scenarios in which the acoustic quality of spoken messages is reduced, other paradigms that more closely simulate everyday listening and other kinds of daily-life acoustic challenge should be explored in future work. Development of digital virtual ‘soundscapes’ would be one such exciting avenue.

Future studies should investigate prosody processing in non-English-speaking patients with dementia, and determine which emotional nonverbal vocal signals are more easily transferred transculturally and cross-linguistically. It will also be important to study patients with other dementia syndromes, including behavioural variant and right temporal variant frontotemporal dementia, in whom impaired emotional prosody perception has previously been identified^65^. It would additionally be interesting to see whether comprehension of noise-vocoded emotional prosody can be modulated pharmacologically, by dopaminergic or cholinergic stimulation^59,66,67^ and/or perceptual learning^54^. This paradigm should also be extended to establish the brain basis and neural mechanisms for comprehending degraded prosodic and other more complex socio-emotional signals in dementia, such as sarcasm^21^. Further, it would be of interest to investigate the extent to which different diagnostic groups rely on specific prosodic cues when perceiving and comprehending emotional prosody. Finally, conversation analysis methods could be employed to explore how these deficits of emotional prosody comprehension under degraded listening conditions impact real-world interactions between people with dementia and their communication partners^68^.

## Conclusions

The findings presented here open a window on a dimension of real-world emotional communication that has often been overlooked in dementia but is particularly pertinent to social cognitive functioning and communication. We currently lack brief, easy-to-administer, ecologically relevant and quantifiable measures of social cognitive and communication function for major dementias, suitable for use in clinical settings. The present work suggests that comprehension of noise-vocoded emotional prosody may be a candidate paradigm for generating measures of this kind.

## Electronic supplementary material

Below is the link to the electronic supplementary material.

Supplementary Material 1

## Data Availability

The datasets generated and/or analysed during the current study are not publicly available due to the stipulation of the institutional ethics approvals covering consent and data collection, but are available from the corresponding author on reasonable request.

## Abbreviations

AD: Alzheimer’s disease
lvPPA: logopenic variant primary progressive aphasia
mIRI: modified Interpersonal Reactivity Index
MMSE: Mini Mental State Examination;
nfvPPA: Nonfluent/agrammatic variant primary progressive aphasia
NHNN: National Hospital for Neurology and Neurosurgery
PALPA: Psycholinguistic Assessment of Language Processing in Aphasia
PPA: Pimary progressive aphasia
RSMS: Revised Self-Monitoring Scale
svPPA: semantic variant primary progressive aphasia;
T-MMSE: telephone Mini Mental State Examination
WASI: Wechsler Abbreviated Scale of Intelligence
