# Supplementary material for: Comprehension of acoustically degraded emotional prosody in Alzheimer’s disease and primary progressive aphasia
Source: Sci Rep. 2024 Dec 28;14:31332. doi: 10.1038/s41598-024-82694-z (PMC11682080; doi:10.1038/s41598-024-82694-z)
Supplement: Supplementary file 1 — Supplementary Material 1 [file 41598_2024_82694_MOESM1_ESM.docx]

**SUPPLEMENTARY MATERIALS: Comprehension of acoustically degraded emotional prosody in Alzheimer’s disease and primary progressive aphasia**

Jessica Jiang, Jeremy CS Johnson, Maï-Carmen Requena-Komuro, Elia Benhamou, Harri Sivasathiaseelan, Anthipa Chokesuwattanaskul, Annabel Nelson, Ross Nortley, Rimona S Weil, Anna Volkmer, Charles R Marshall, Doris–Eva Bamiou, Jason D Warren, Chris JD Hardy

**Assessment of peripheral hearing function**

Using a GSI Audiostar Pro^TM^ audiometer (<https://www.guymark.com/product-information/hearing-assessment/audiometers/clinical/gsi-audiostar-pro>) and calibrated GSI Audiostar Pro headphones with noise-reducing ear-cups, steady tones of 250, 500, 1000, 2000, 4000 and 8000Hz were presented separately to each of the participant’s ears. At each frequency, the participant indicated (verbally or by gesture) when they first heard a noise. The decibel hearing level was set typically at 50 dB, with decreases of 10 dB if they could hear the tone, and increases of 5dB if they could not. The average threshold in dB hearing level across the 500, 1000, 2000 and 4000 Hz frequencies most relevant to speech processing [69] in both ears were calculated, and the minimum was taken as the better ear average.

**Synthesis of noise-vocoded stimuli**

Raw speech recordings were noise-vocoded in Praat (<https://www.fon.hum.uva.nl/praat/>) using a script written by Chris Darwin: <http://www.lifesci.sussex.ac.uk/home/Chris_Darwin/Praatscripts/Shannon> to generate speech stimuli with 6, 12, and 18 frequency bands (‘channels’). Log spacing for each channel number was calculated between 50 and 8000Hz using the logspace function in Matlab R2014a. Average (rms) stimulus intensity was fixed in Matlab to be constant for all stimuli, and all stimuli were windowed with 20ms onset-offset temporal ramps to prevent click artefacts.

**Remote testing procedure**

Twenty-five participants (four healthy controls, eight patients with Alzheimer’s disease (AD), five with logopenic variant primary progressive aphasia (lvPPA), five with nonfluent/agrammatic variant primary progressive aphasia (nfvPPA) and three with semantic variant primary progressive aphasia (svPPA)) were assessed remotely via a video link, due to the COVID-19 pandemic. We have detailed the design and implementation of our remote neuropsychological assessment protocol elsewhere [42].

An initial session was conducted via Zoom to accustom participants to the remote testing format, check the screen and sound sharing options on Zoom, and that the quality of the participant’s internet connection was acceptable. Participants were permitted to use their preferred technology interface (computers/tablets; smartphones were not allowed, to ensure good screen visibility). Remote assessments were scheduled to ensure testing could be completed in a quiet environment with minimal distractions, and the device volume was set to a comfortable level by each participant or their caregiver. The experiment was implemented onto Labvanced for administration [70]. Most participants listened in free field, over their device speakers (only a few used headphones); of the seven healthy control participants who performed the experiment both in person and remotely, only one used headphones for the remote session.

A further audibility check was used for each participant through the Bamford-Kowal-Bench [71] list. The spoken BKB sentences were delivered online using Labvanced; a perfect score of the final 3 items on the test was required to go on to the remote testing session proper (this allowed each participant and/or caregiver to manually adjust the volume to a comfortable level for clear audibility). Most participants (97%) performed at ceiling; none was rejected based on their BKB performance.

Each patient’s caregiver was required to be available during each research session in case of any problems using the equipment. In practice, however, no major technological issues arose during the remote testing sessions.

The remote experimental procedure was otherwise identical to the in-person procedure.

Performance profiles in seven healthy control participants who performed the experiment both in person (via headphones) and remotely (in free-field) were very similar (see Figure S3), justifying combining participants tested in person and remotely in the main analysis.


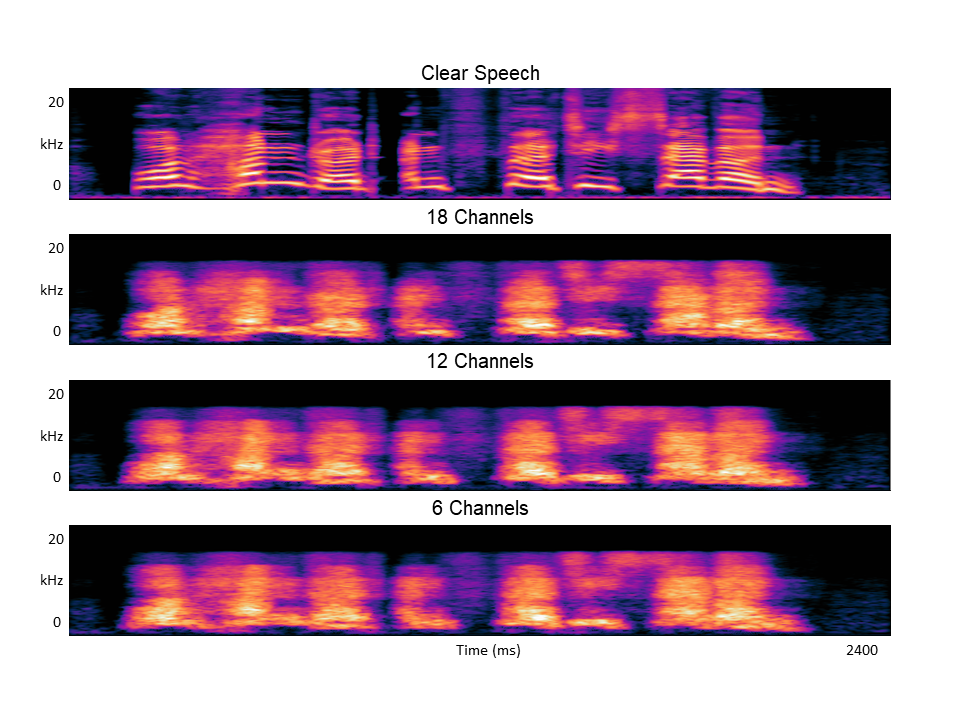


**Figure S1.** **Spectrograms of clear (natural) speech and noise-vocoded speech resampled with differing numbers of discrete frequency bands (18 channels, 12 channels, and 6 channels).** The verbal message in each case is the spoken number ‘seven hundred and fifty-six’. The spectrogram provides a visual indication of how the energy in different frequency bands of the speech signal (plotted on y axis) changes over time (plotted on x-axis). Reducing the number of channels (frequency bands) progressively reduces the amount of spectrotemporal fine structure and thus degrades the intelligibility of the speech signal.


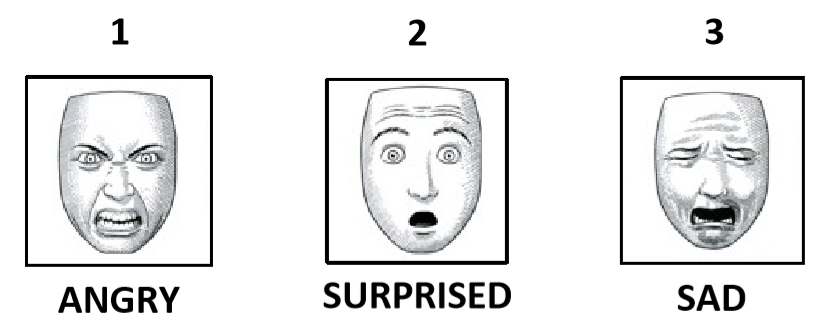


**Figure S2.** **Sex-neutral facial expressions cue card used in experiment.** The cue card displays the three emotions (one target, two foils) on masks (i.e., sex-neutral faces), each labelled with their corresponding emotion.


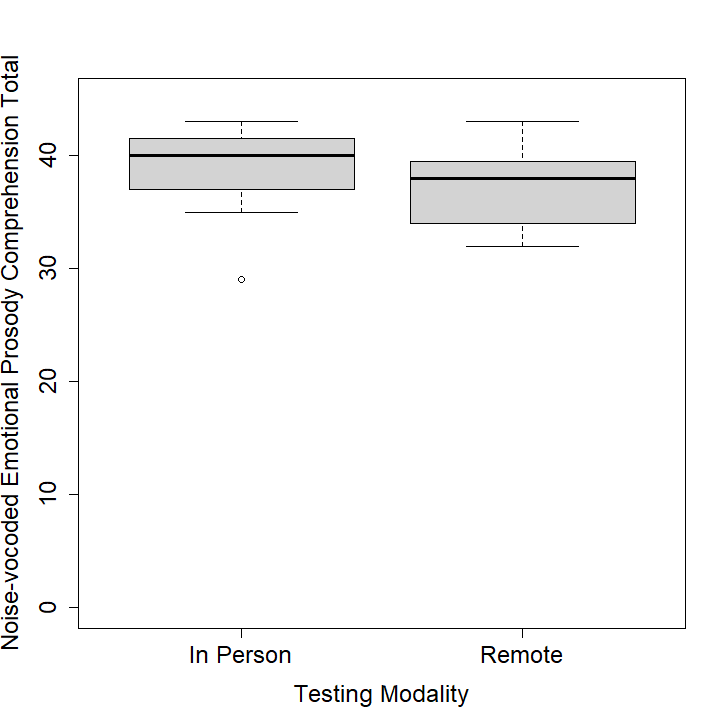


**Figure S3. Comparison of healthy older controls’ performance on comprehension of noise-vocoded emotional prosody for in-person vs remote testing sessions.** Seven healthy older control participants performed the noise-vocoded emotional prosody comprehension task both face-to-face (F2F) at the research centre and remotely in their home environments, approximately 20 months later. Box plots show the mean and standard deviation (whiskers indicate the full range) of speech intelligibility thresholds in each session. The differences between the two sessions were non-significant (F2F mean = 38.43; Remote mean = 37.14; p = 0. 610).


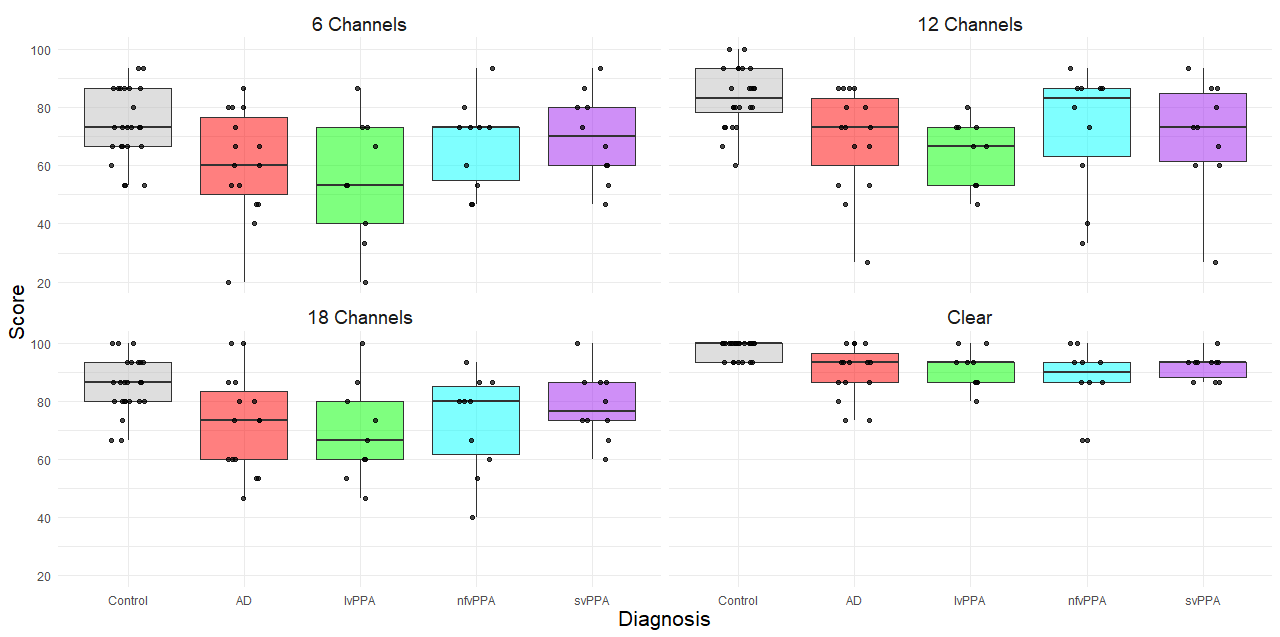


**Figure S4. Emotion identification performance for all clear and noise-vocoded conditions for each participant group.** The line within each box indicates the median, with the boxes indicating the interquartile range. AD, patient group with Alzheimer’s disease; Control, healthy older individuals; lvPPA, patient group with logopenic variant primary progressive aphasia; nfvPPA, patient group with nonfluent/agrammatic variant primary progressive aphasia; svPPA, patient group with semantic variant primary progressive aphasia.


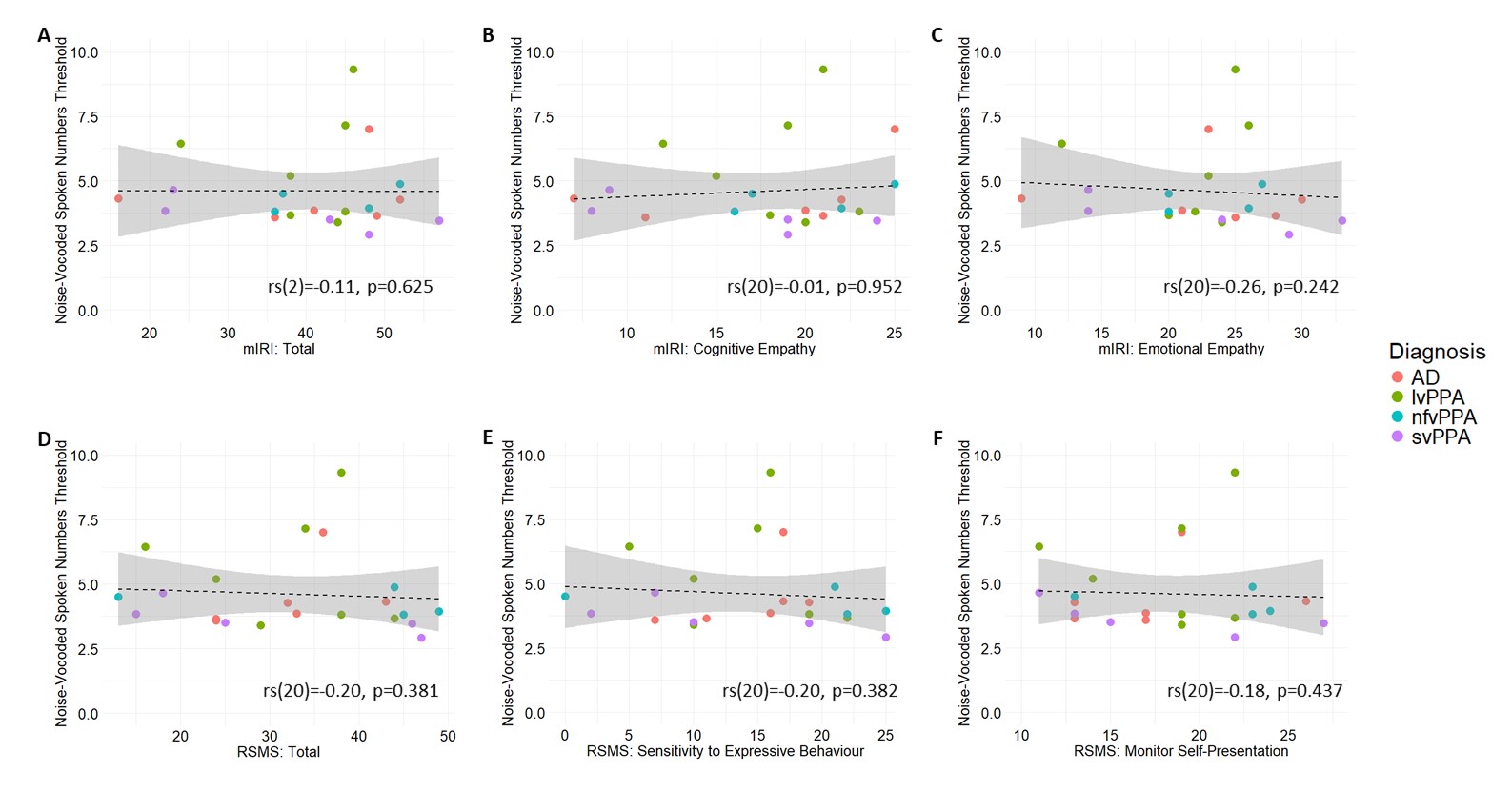


**Figure S5. Correlation plots of noise-vocoded spoken number repetition with measures of social cognition across the patient cohort.** The Figure shows how different standard measures of social cognition were correlated with 50% threshold intelligibility (accuracy of repetition) of noise-vocoded spoken numbers (as described in [39]) across syndromic groups, as follows: (A) correlation with the full modified Interpersonal Reactivity Index (mIRI); (B) correlation with the cognitive empathy subscale in mIRI; (C) correlation with the cognitive empathy (perspective taking) subscale in mIRI; (D) correlation with the full revised self-monitoring scale (RSMS); (E) correlation with the sensitivity to socio-emotional expressiveness RSMS subscale; (F) correlation with the monitoring self-presentation RSMS subscale. Spearman’s rank and p-value shown alongside each correlation line, no correlations were significant. Dots represent each participants’ performance, with colours representing each syndromic diagnosis, as coded in the key (right). AD, patient group with Alzheimer’s disease; lvPPA, patient group with logopenic variant primary progressive aphasia; mIRI, modified Interpersonal Reactivity Index; nfvPPA, patient group with nonfluent variant primary progressive aphasia; RSMS, revised self-monitoring scale; svPPA, patient group with semantic variant primary progressive aphasia.
